# SurvNet: A Novel Deep Neural Network for Lung Cancer Survival Analysis With Missing Values

**DOI:** 10.3389/fonc.2020.588990

**Published:** 2021-01-20

**Authors:** Jianyong Wang, Nan Chen, Jixiang Guo, Xiuyuan Xu, Lunxu Liu, Zhang Yi

**Affiliations:** ^1^ Machine Intelligence Laboratory, College of Computer Science, Sichuan University, Chengdu, China; ^2^ Department of Thoracic Surgery, West China Hospital and West China School of Medicine, Sichuan University, Chengdu, China

**Keywords:** survival analysis, prognosis prediction, deep neural networks, multi-task learning, missing value

## Abstract

Survival analysis is important for guiding further treatment and improving lung cancer prognosis. It is a challenging task because of the poor distinguishability of features and the missing values in practice. A novel multi-task based neural network, SurvNet, is proposed in this paper. The proposed SurvNet model is trained in a multi-task learning framework to jointly learn across three related tasks: input reconstruction, survival classification, and Cox regression. It uses an input reconstruction mechanism cooperating with incomplete-aware reconstruction loss for latent feature learning of incomplete data with missing values. Besides, the SurvNet model introduces a context gating mechanism to bridge the gap between survival classification and Cox regression. A new real-world dataset of 1,137 patients with IB-IIA stage non-small cell lung cancer is collected to evaluate the performance of the SurvNet model. The proposed SurvNet achieves a higher concordance index than the traditional Cox model and Cox-Net. The difference between high-risk and low-risk groups obtained by SurvNet is more significant than that of high-risk and low-risk groups obtained by the other models. Moreover, the SurvNet outperforms the other models even though the input data is randomly cropped and it achieves better generalization performance on the Surveillance, Epidemiology, and End Results Program (SEER) dataset.

## Introduction

In clinical research, the development of effective survival analysis methods for censored data is always required to evaluate the relationship between the risk factors and event of interest (1, 2). It has been widely applied to modeling the prognosis of cancers to help to optimize and improve cancer treatment (3–6).

Lung cancer is one of the most heterogeneous cancers and has distinct prognoses. A great deal of work has been conducted on lung cancer prognostic prediction in recent decades, among which the series of tumor node metastasis classification (TNM classification) for lung cancer is the most famous one (4, 7, 8). It has been the guideline for clinical treatment. In the eighth edition of TNM classification for lung cancer, the five-year survival rate of the IB-IIA stage ranged from 65 to 73%, which is relatively high. However, in the practice, many IB-IIA stage patients present with a recurrence and die within five years after treatment. Distinguishing the IB-IIA stage patients with a high risk of recurrence and death from low-risk patients is worthwhile for guiding further treatment and may improve the lung cancer prognosis. Additionally, the clinicopathologic variable used in TNM classification is limited for personalized prediction for different patients and it is limited to integrate new variables into the existing prognosis models (9). There is a great need for a new survival analysis method to establish fine-grained prognoses for individual patients with IB-IIA stage lung cancer for more accurate individual prediction by integrating an expanding number of prognostic factors.

Cox (2, 10, 11) proportional hazards regression is one of the most well-known survival analysis methods. It has been implemented in many famous software toolboxes and been widely used in many prognosis prediction tasks (8), such as TNM classification for lung cancer (4, 7, 8). The Cox proportional hazards model is semi-parametric and is subject to a linear model (12). It makes an important assumption about the hazard function, which is that covariances that affect the hazard rate are independent. However, in practice, the relationship between variables and the outcome is complex and unknown and there may be interactions among variables (13). Deep neural networks (DNNs) is apparent to be a promising method to solve these problems.

The DNN is a class of biologically inspired computational models towards artificial intelligence. It has been proven that DNNs can approximate any non-linear function when provided with sufficient neurons. Generally, a DNN can be a very complex non-linear model and learn latent features from data directly (14). It has achieved many impressive results in various applications, such as image classification (15, 16), natural language processing (17–20), and biomedical analysis (14, 21–23) in addition to survival analysis (1, 2, 7, 11, 24–26).

Generally, most of DNN based methods for survival analysis could be divided into two paradigms. The first is to formulate the survival analysis as a classification problem to evaluate survival probability at different fixed time points (9, 27, 28). In (27), neural networks were used to improve the prediction accuracy of the five-year survival of patients with breast cancer. Lundina et al. demonstrated that a neural network model trained on some prognostic factors can accurately predict specific 5-, 10-, and 15-year breast cancer survival (9).

The other paradigm is to extend Cox regression with DNNs, in which DNNs are used to extract the features of the patient and trained using Cox-like cost function with the gradient-based method. In (12), the authors proposed the Cox-Net model for prognosis prediction on high-throughput omics data and implemented it with the Theano math library in Python to achieve an efficient computational time using GPUs. Huang et al. modified the Cox-Net method to use multi-omics survival analysis learning on breast cancer (26). Moreover, (16), DNN was applied to cardiac motion analysis for human survival prediction and outperformed the traditional Cox models. However, Cox-Net inherited the limitation of Cox models, which is that it was not designed to estimate the probability of survival at a fixed time. Therefore, it is necessary to study how to design a unified model that integrates the good properties of the aforementioned two paradigms to improve the performance.

Moreover, the dataset used in survival analysis commonly contains incomplete data with missing values in practice. In many cases, most of the patients with missing values are excluded (2, 29). Omitting patients with missing values limits the number of patients to train the prognosis model and may introduce substantial biases in the study, whereas using patients with missing values may harm the performance. The learning of the latent feature of incomplete data with missing values in survival analysis by using DNNs should be evaluated further.

To address the aforementioned problems, in this study, we propose a multi-task based neural network model, SurvNet, for survival analysis of real-world datasets of patients with IB-IIA stage lung cancer. The main contributions are as follows:

- An input reconstruction mechanism cooperating with incomplete-aware reconstruction loss is proposed in the SurvNet for latent feature learning of incomplete data with missing values.
- A context gating mechanism is proposed in the SurvNet to bridge the gap between survival classification and Cox regression for prognosis prediction.
- The proposed SurvNet model is trained in a multi-task learning framework to jointly learn across three related tasks: input reconstruction, survival classification, and Cox regression.
- A new real-world dataset is collected to evaluate the performance of the prognosis prediction models for IB-IIA stage non-small lung cancer.

The proposed method is compared with the traditional Cox model and Cox-Net in the experiments. The experiment results demonstrate that the proposed SurvNet outperforms the other models with a much higher concordance index (C*_index_*). The difference between high and low risk groups obtained by SurvNet is more significant than that of high and low risk groups obtained by the other models. Furthermore, it achieves better performance on incomplete data with missing values and better generalization performance on Surveillance, Epidemiology, and End Results Program (SEER) dataset.

## Materials and Methods

### Datasets

**Figure 1:**
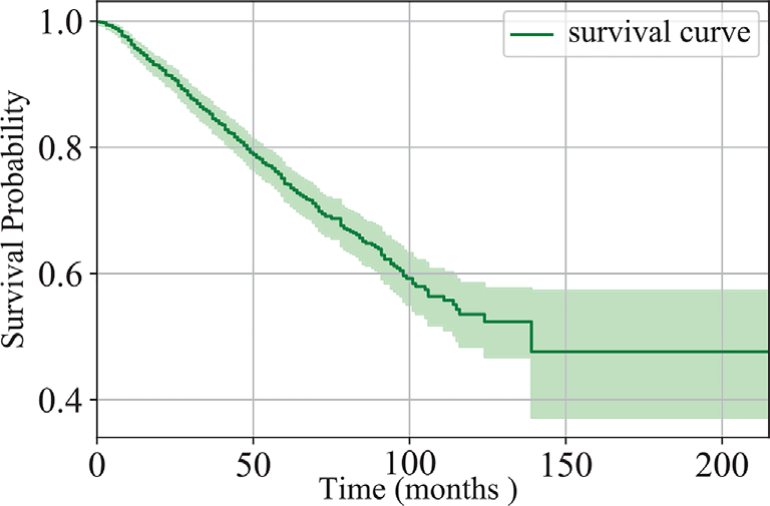
The Kaplan-Meier estimation of the datasets presented in this study.

In this study, we collected the data of 1,280 patients with IB-IIA stage non-small cell lung cancer at West China Hospital, from 2005 to 2018. There are 1,137 patients remaining after the exclusion of patients with unknown survival time. Of the 1,137 patients, 346 died and the others are missing follow-up or still alive. The survival time of the patients is in the range of (1,215) months. **Figure 1** shows the Kaplan-Meier estimation of the dataset. Clearly, the five-year survival probability of the patients at the IB-IIA stage is relatively high. However, 42, 97, 160, 219, and 263 patients died in 1-, 2-, 3-, 4-, and 5-year respectively. Distinguishing the IB-IIA stage patients with a high risk of recurrence or death from low-risk patients is worthwhile for guiding further treatment and improving the non-small lung cancer prognosis.

In this study, nine clinicopathologic variables are taken into account for prognosis prediction. The distribution of the variables in the final patient series used in the study is shown in **Table 1**. In practice, it is difficult to ensure all of the variables were recorded for each patient. As illustrated in **Table 1**, there are lots of missing values (denoted as “unknown”). Of 1,137 patients, 961 contains missing values (at least one of the clinicopathologic variables is missing). It brings a great challenge for prognosis prediction models.

**Table 1:** Distribution of clinicopathologic variables in our datasets of patients with IB-IIA stage lung cancer. Missing values are denoted as “unknown”.

Clinicopathologic variables	Value	Num of patients
Age	*Range* (18–86)	1,137
Tumor Size	*Range* (0.3–5) **Unknown**	778 359
Sex	*Female* *Male*	670 467
Tumor Location	*Left Upper* *Left Lower* *Right Upper* *Right Middle* *Right Lower*	287 186 367 87 210
Differentiation	*Lower* *Middle or High* **Unknown**	501 542 94
Cancer Type	*Adenocarcinoma* *Squamous carcinoma*	843 294
Lymp node management	*Dissection* *Biopsy or No Management* **Unknown**	1,015 97 25
Pleura Immersion	*Yes* *No* **Unknown**	656 87 394
Operation	*Lobectomy* *Sub Lobectomy*	53 1,084

Of nine clinicopathologic variables, age and tumor size are continuous variables, whereas the others are encoded using discrete values, for example, –1 for females and 1 for males. The missing values are filled with zeros. Thus, the clinicopathologic variables of each patient are represented by a 9 dimension vector. Formally, the proposed dataset can be formulated into a set of triplets {(*x_i_*, *s_i_*, *t_i_*) |*i* = 1, 2, ^…^, *n*}, where *n* is the number of the patients, *x_i_* ∈ *R* ^9^ is a vector of 9 clinicopathologic variables that describes the *i*-th patient, *s_i_* is the patient’s end state, that is, 1 for dead or 0 for alive, and *t_i_*is the patient’s survival time.

### Problem Definition

In this study, the aim is to dichotomize the patients in the dataset into high and low risk groups according to their prognosis index. This can be formulated as:

(1) $$d_{x}=\{\begin{array}{l} 1,if\;p_{x}>PI\\ 0,if\;p_{x}<=PI \end{array}$$

where PI is a constant and the prognosis index *p_x_* is calculated by

(2) $$p_{x}=F\left(x\right)$$

where *F* is generally a complex non-linear function. Thus, the core task of the prognosis prediction task is to determine a suitable function *F*.

However, it is difficult to estimate the prognosis index function *F* for fine-grained prognosis prediction of IB-IIA stage lung cancer. There are three challenges:

- For most patients, the end event (death) has not yet happened. It is known as censoring. In other words, we could not get actual survival times for these patients.
- Most of the patients contain at least one missing value. Omitting patients with missing values may bias the result, whereas using patients with missing values may harm the performance. It is a great obstacle for machine learning methods (30, 31).
- The distinguishing feature is difficult to learn for patients with IB-IIA stage lung cancer.

### Multi-Task Based SurvNet for Prognosis Prediction

**Figure 2:**
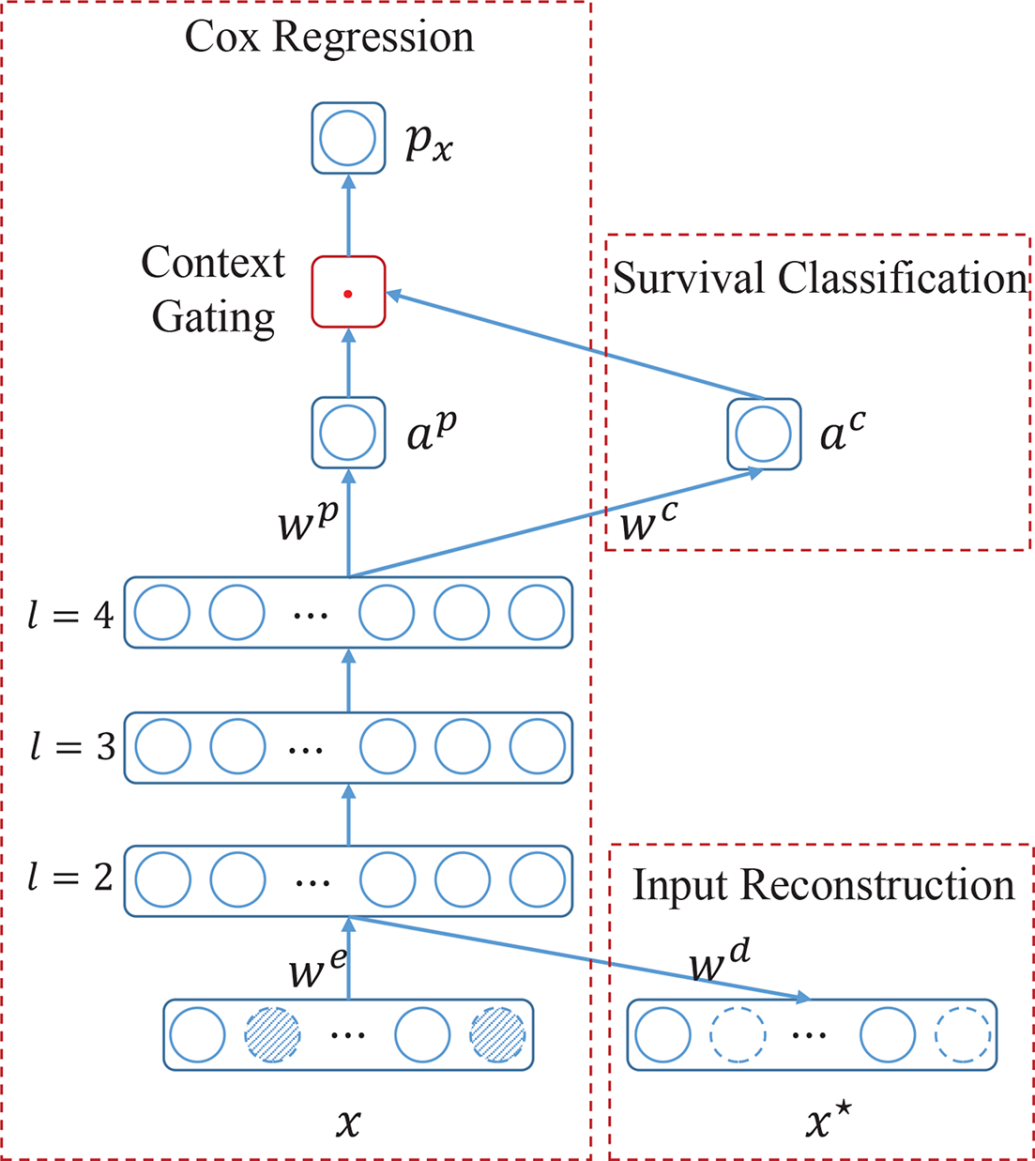
The architecture of the proposed SurvNet for prognosis prediction. SurvNet consists of a main module, i.e., Cox regression, and two auxiliary modules, i.e., survival classification and input reconstruction. *x* and x* are input and reconstructed input, respectively. *w^e^*, *w^d^*, *w^p^*, *w^c^* are trainable weight parameters. *l* is the layer index. *α^p^*, and *α^c^* are activations of neurons. *p_x_* is the prognosis index. ‘.’ denotes a multiplication operation. Dotted circles filled with oblique lines denote the missing values.

To overcome the aforementioned difficulties, in this study, a novel multi-task based neural network namely SurvNet is proposed for the prognosis prediction of IB-IIA stage lung cancer. As **Figure 2** illustrated, the proposed SurvNet consists of three modules: Cox regression module, survival classification module, and input reconstruction module. Cox regression module is the main backbone of SurvNet and is used to represent the Function *F* for prognosis prediction. Survival classification module and input reconstruction module are auxiliary modules that aim to improve the performance of SurvNet for fine-grained prognosis prediction on incomplete data with missing values.

#### Input Reconstruction Module

The missing value is a common phenomenon in survival analysis. The suitable method to deal with the missing values is always desired to improve the performance of prognosis prediction models. In the proposed SurvNet, we use zeros to fill the missing values and then learn the latent feature of incomplete data by input reconstruction.

Given an input *x*, it is first encoded into latent feature vector *a* ^2^ (the output of layer 2). Then *a* ^2^ would be feed into input reconstruction module to be decoded into x*, a reconstruction of input *x*. Formally, it can be formulated as:

(3) $$\{\begin{array}{l} a^{2}=f\left(w^{e}x\right)\\ x^{*}=w^{d}a^{2}, \end{array}$$

where *w^e^* and *w^d^* are encoder and decoder weights, *x* and x* are input and reconstruction respectively. *f* denotes the non-linear activation function. Actually, the input layer, 2^nd^ layer, and the input reconstruction layer composes an autoencoder network (See **Figure 2**).

Generally, mean square error (MSE) is used to make x* approximate *x* as accurately as possible. However, it is not suitable for input with missing values since we do not know the true values at such locations and do not want to reconstruct a new vector with missing values too. To address this problem, an incomplete-aware cost function is proposed to learn the latent feature of incomplete data with missing values.

Let the binary vector *r* denote the locations of missing values in input *x*:

(4) $$r\left(j\right)=\{\begin{array}{l} 0,thej–th\;element\;of\;x\;is\;missing\\ 1,otherwise \end{array}$$

The propsoed incomplete-aware cost function is formulated as:

(5) $$J_{re}=\frac{1}{2n}\sum_{i=1}^{n}{\left||r_{i}\cdot \left(x_{i}^{*}-x_{i}\right)|\right|}_{2}$$

where *i* is the sample index and “·” denotes the elementwise product.

#### Survival Classification Module

To fully *x_i_*, utilize the relationship among the input *variables x_i_*, end state *s_i_* and survival time *t_i_*, we introduce an auxiliary survival classification module to the SurvNet. The output of this module *a* ^c^ denotes the probability of the patient living over *T* years or not. In other words, it learns a patient’s survival probability at some fixed time point *T*. Formally, *a* ^4^ could be calculated as:

(6) $$a^{c}=\sigma \left(w^{c}a^{4}\right)$$

where *w* ^c^ and *a* ^4^ are weight connection and output of layer 4, and σ(*z*) = 1/(1+ exp(–*z*)). In this study, layer 3 is a batch norm layer. *a* ^4^ could be calculated as:

(7) $$\{\begin{array}{l} a^{4}=\tanh\left(w^{3}a^{3}\right)\\ a3=\frac{a^{2}-E\left[a^{2}\right]}{Var\left[x\right]+\in }, \end{array}$$

where *w* ^3^ is weight connection and tanh(*z*) = (exp(–z) – exp(*z*))/(exp(–*z*) + exp(*z*)). *E* and *Var* denote the expectation and variance, respectively. ϵ is a small constant.

In this manuscript, the learning of survival classification module could be formulated as a binary classification task, which aims to minimize the following cost function:

(8) $$J_{c}=\sum_{i}^{n}\delta_{i}\left(d_{i}\cdot \log\left(a^{c}\right)+\left(1-d_{i}\right)\cdot \left(\log\left(1-a^{c}\right)\right)\right).$$

where *d_i_* is the survival state of the patient *i*, which is defined as

(9) $$d_{i}=\{\begin{array}{l} 1,if\;t_{i}>T\\ 0,if\;t_{i}<\;T\;and\;s_{i}==1, \end{array}$$

and *δ_i_*denotes whether a patient is a valid sample classification task and it is defined as

(10) $$\delta_{i}=\{\begin{array}{l} 1,if\;t_{i}<T\;and\;s_{i}==0\\ 0,otherwise. \end{array}$$

It means that patients censored before *T* are ignored.

#### Cox Regression Module

This is the main backbone of the proposed SurvNet. As **Figure 2** illustrated, it consists of several successive feedforward layers, such as fully connected layer, batch normalization (32), and dropout layer, and a new context gating submodule that does not exist in the traditional Cox-Net (12).

Given an input *x*, the high-level representation *α^L^* could be calculated layer by layer. Specifically, the activation *α^p^* is computed as

(11) $$a^{p}=w^{p}a^{L}.$$

In the traditional Cox-Net (12). *α^p^* would be expressed as log hazard ratio in Cox regression. However, in the proposed SurvNet, the distribution of log hazard ratio *α^p^* is adjusted by survival probability *α^c^* by using context gating mechanism:

(12) $$p_{x}=a^{p}\cdot a^{c}.$$

Then, we take *p_x_* as log hazard ratio in Cox regression and use the following log partial likelihood for Cox regression:

(13) $$J_{cox}=\sum_{i:s_{i}=1}^{n}\left(p_{x_{i}}-log\left(\sum_{j:t_{j}\leq t_{i}}p_{x_{j}}\right)\right),$$

where *i* and *j* are sample indexes.

The context gating mechanism is inspired by the attention mechanism where the input is adjusted by the attention coefficient. It is notable that the proposed context gating mechanism bridges the gap between Cox regression and survival classification to improve the performance. On the one hand, in the Cox regression, it is supposed that the larger the survival time, the larger the prognosis index. It reveals that the prognosis indexes of patients that are alive at some fixed time point *T* should be larger than the patients that died at that time point. On the other hand, the survival classification aims to predict the survival state of a given patient at a fixed time point *T*. As Equation (12) illustrates, the survival prediction *α ^c^* serves as a context coefficient that adjusts the hazard ratio *α ^p^* automatically thus to produce a better prognosis index that has good distribution at time point *T*.

#### Multi-Task Learning

To train the proposed SurvNet, three learning tasks are optimized synchronously. The final cost function could be formulated as:

(14) $$J=\alpha J_{cox}+\beta J_{C}+\gamma J_{re},$$

where α, β, and γ are the coefficients that balance the Cox regression, survival classification, and input reconstruction tasks. By using gradient-based algorithms, the (local) minimal of cost function *J* could be found iteratively.

## Experiments

### Evaluation Metrics

To evaluate the performance of the proposed model, two metrics were used. One is the Harrell’s concordance index (C_index_), which is valued from 0 to 1. It is an extension of the area under the receiver operating characteristic curve to censored time-to-event data (16, 26).

Generally, it is defined as

(15) $$C_{index}=\frac{\Sigma_{i,j}\;s_{i}\cdot I(p_{i},\;p_{j})\cdot I\left(t_{i},t_{j}\right)}{\Sigma_{i,j,}\;s_{i}\cdot I\left(t_{i,}\;t_{j}\right)}$$

Where *i* and *j* are sample indexes. *s*, *t*, and *p* are end state, survival time and hazard ratio of a given sample, respectively. *I*(*z*_1_, *z*_2_) is defined as:

(16) $$I\left(z_{1},z_{2}\right)=\{\begin{array}{l} 1,if\;z_{1}<z_{2}\\ 0,otherwise. \end{array}$$

The other metric is the survival analysis with the log-rank test. Kaplan-Meier survival curves are generated by dichotomizing all patients in the testing dataset into low-risk and high-risk groups *via* the median hazard ratio. The corresponding log-rank p-value indicates the ability of the model to differentiate two risk groups. The lower the p-values, the better the model performance.

### Running Configuration

#### Datasets

To train the prognosis models, the presented dataset was randomly split into train set (682 patients), validation set (227 patients), and test set (228 patients). Furthermore, we also obtained a SEER dataset (9,534 patients) by selecting the IB-IIA stage lung cancer patients from SEER to test the generalization performance of the models.

#### Models

The proposed SurvNet was compared with the traditional Cox proportional hazards model and neural network extended Cox model (Cox-Net). For a fair comparison, the Cox-Net shared the same architecture with the Cox regression module in SurvNet except for the context gating module. The network settings is presented in **Table 2**. Besides, we set *T* = 36 for SurvNet and the coefficients α, β, and γ were set to 0.2, 1, 3, respectively. The RMSProp (33) with default learning parameters in Pytorch was used as the optimizer and the weight decay was set to 0.00001. All of the networks run 100 epochs with batch size 64. For each run, the weight parameters that achieved the best C*_index_* on the validation dataset were used to evaluate the performance of the model on the test dataset.

**Table 2:** Network settings for Cox-Net and SurvNet.

Layer	Neurons
1	9 (input)
2	64 (tanh)
3	Batch normalization
4	32 (tanh)

### Performance on Our Dataset

**Figure 3:**
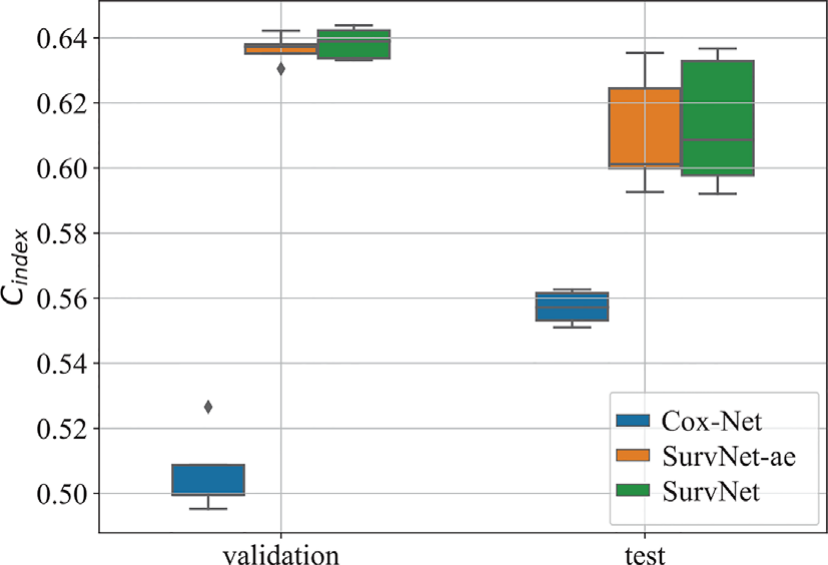
The boxplot of the C*_index_* on the validation dataset and test dataset. “SurvNet-ae” denotes the SurvNet without survival classification module.

To eliminate the influence of initial values of neural networks, we run Cox-Net, SurvNet, and SurvNet-ae (SurvNet without survival classification module) five times. For each running, the model with the highest C*_index_* on the validation dataset is selected to evaluate the performance on the test dataset. The boxplot of the C*_index_* is presented in **Figure 3**. It demonstrates that the proposed SurvNet with and without survival classification module outperformed the Cox-Net significantly by the using input reconstruction module to learning the latent feature of incomplete data with missing values. And the proposed survival classification module further improves the network’s performance. Besides, the best C_i_ *_ndex_* of traditional Cox model, Cox-Net, and the proposed SurvNet are 0.5612, 0.5627, 0.6367, respectively. The proposed SurvNet outperforms the other models significantly.

**Figure 4:**
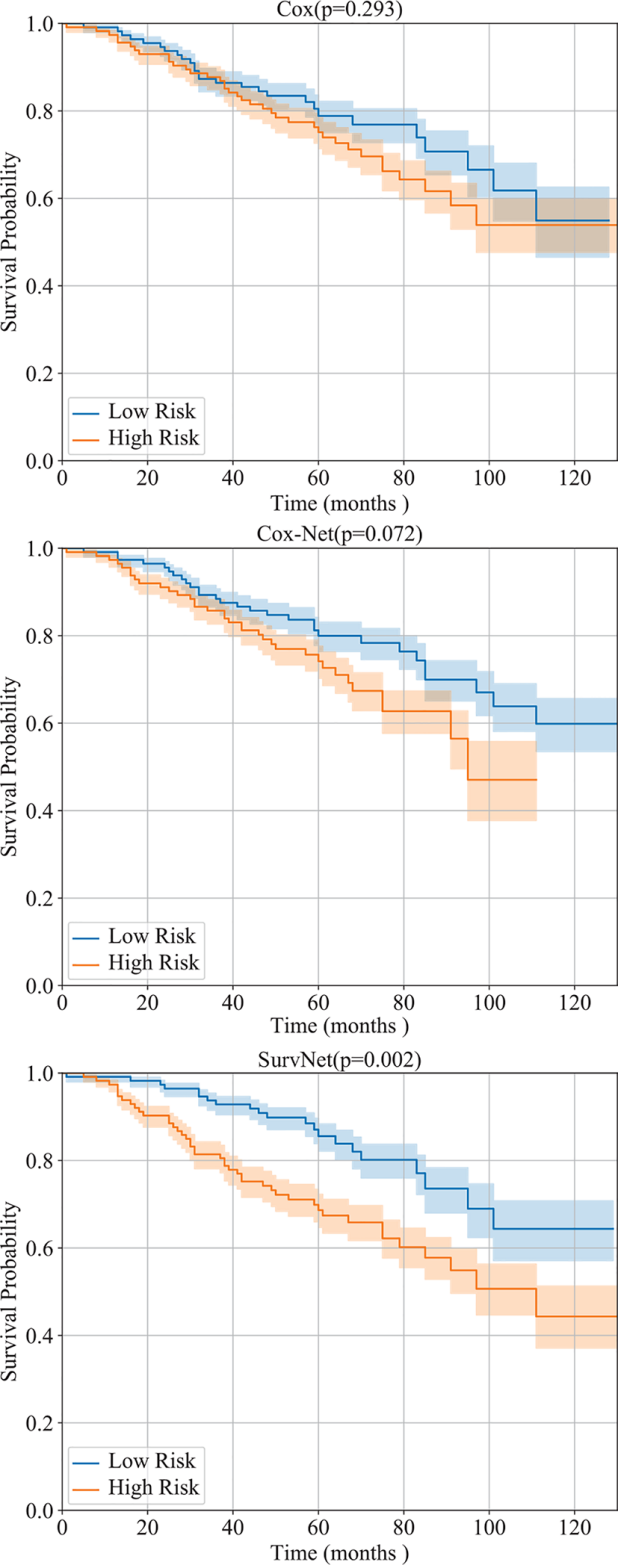
Performance of the Cox model, Cox-Net, and the proposed SurvNet on our test dataset. The Kaplan-Meier estimation (with 95% confidence intervals) of high risk and low risk groups are shown and the log-rank test was performed to compare survival curves between two groups.

Furthermore, by interpreting the outputs of the models as the log hazard ratio, two groups (high risk and low risk) are obtained by using Eq. (1) where *PI* was set to the median of log hazard ratios. The Kaplan-Meier estimation of the Cox model, Cox-Net and the proposed SurvNet on the test dataset are presented in **Figure 4**. The log-rank *p*-values (the lower the better) of the three methods are 0.293, 0.072, 0.002. It is obvious that the difference between high and low risk groups obtained by neural network based models is more significant than that of high and low risk groups obtained by the Cox model. Moreover, the difference between high and low risk groups obtained by SurvNet is most significant. It demonstrates that the proposed SurvNet achieves the best performance.

**Figure 5:**
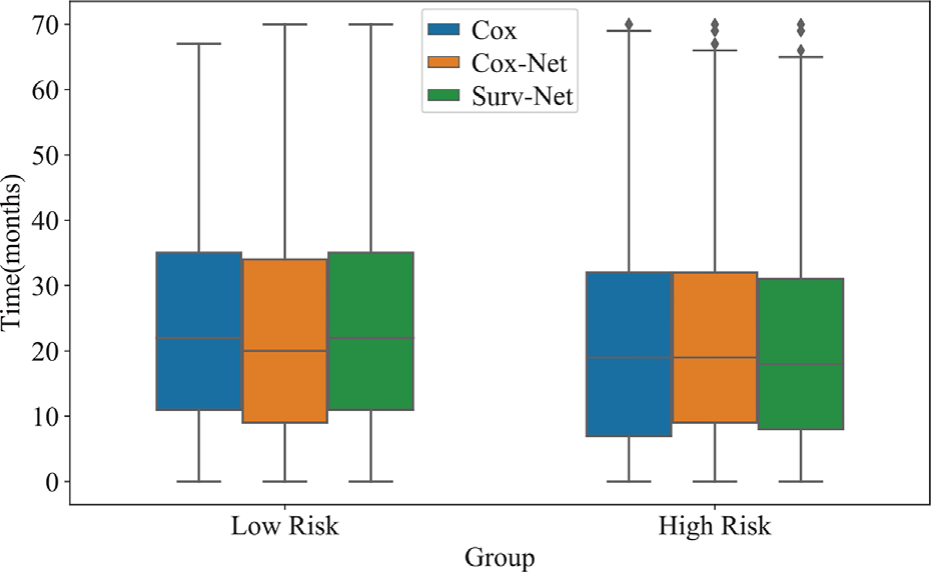
The distribution of survival time of high-risk group and low risk group obtained by three models.

In addition, the distribution of survival times of patients in each group is presented in **Figure 5**. The proposed SurvNet achieves the largest median survival time for low risk group and the lowest median survival time for high risk group. It demonstrates that the proposed method improves the performance of fine-grained prognosis prediction for IB-IIA stage non-small cell lung cancer.

### Robustness on Missing Values

To further evaluate the robustness of prognosis models on incomplete data with missing values, we randomly zeroed the values of the input vector in the test dataset with drop probability *dp* and then evaluated the performance of the trained models. For each drop probability *dp*, we run each model 100 times.

**Figure 6:**
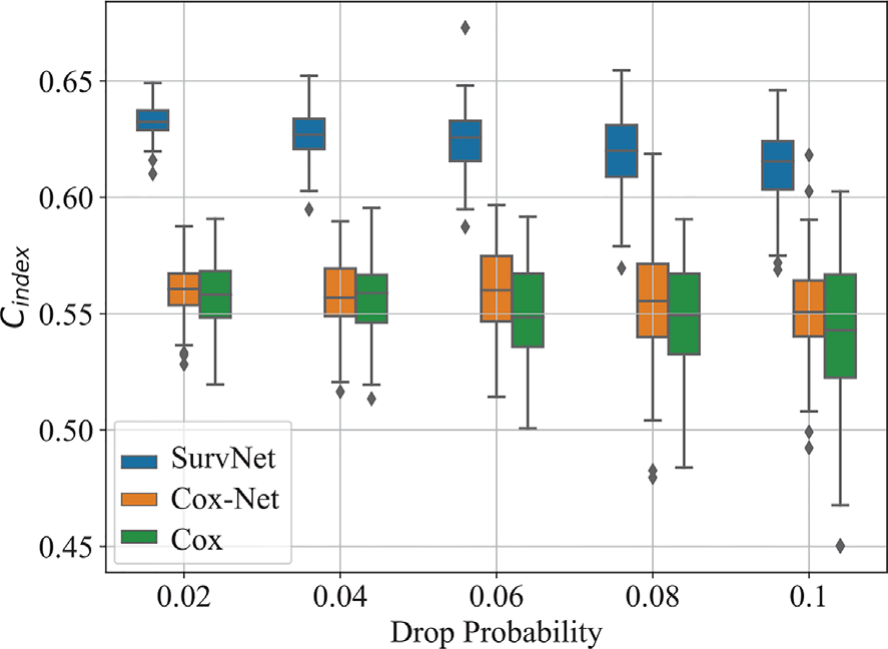
The boxplot of the *C_index_* on the test dataset with different drop probabilities.

The boxplot of C*_index_*is illustrated in **Figure 6**. As drop probability gets large, the performance of the three models gets worse. Notably, the proposed SurvNet performed more stable and the C*_index_*of the SurvNet is always larger than that of the Cox model and Cox-Net significantly.

### Generalization Performance on SEER Dataset

The generalization performance is an important measurement of prognosis models. SEER dataset has been widely used in the literature. In this study, we focused on the IB-IIA stage non-small lung cancer and obtained a dataset of 9,534 patients.

**Figure 7:**
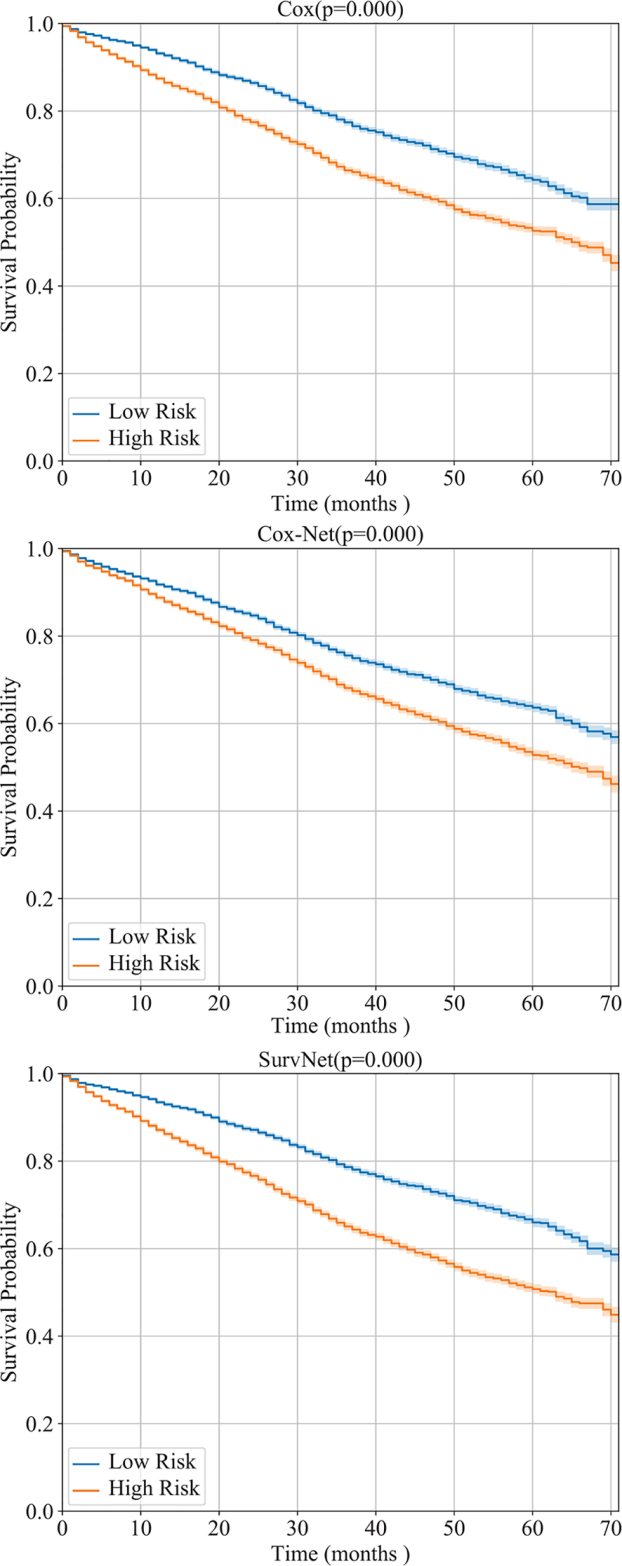
The generalization performance of Cox model, Cox-Net, and the proposed SurvNet on SEER dataset. For each model, the Kaplan-Meier estimation (with 95% confidence intervals) of high risk and low risk groups are shown and the log-rank test was performed to compare survival curves between two groups.

We evaluated the models, which have been trained using our dataset, on the obtained SEER dataset. The C*_index_* of Cox model, Cox-Net, and SurvNet are 0.5955, 0.5617, and 0.6003, respectively. Besides, as the Kaplan-Meier estimation presented in **Figure 7** shows, the difference between high and low risk groups obtained SurvNet is more significant than that of high and low risk groups obtained by other two models. The proposed SurvNet achieves better generalization performance than the Cox model and Cox-Net.

## Conclusion

Prognosis prediction for IB-IIA stage lung cancer is important for improving the accuracy of the management of lung cancer. In this study, a new real-world dataset is collected and a novel multi-task based neural network, SurvNet, is proposed to further improve the prognosis prediction for IB-IIA stage lung cancer. In the proposed SurvNet, the input reconstruction module overcomes the problems by missing values and the proposed context gating mechanism could bridge the gap between Cox regression and survival classification. By training in a multi-task framework, the proposed SurvNet outperforms the traditional Cox model and Cox-Net significantly. It achieved higher C*_index_*s and lower *p*-values on the proposed dataset and better generalization performance on the SEER dataset. It is apparent to be a promising method for survival analysis tasks. A limitation of the proposed SurvNet may lie on the survival classification module which just considers survivals on some fixed time point rather than a set of non-overlap time intervals. Future work will be focused on how to integrate survival classification module that classifies the survivals into a set of time intervals with the Cox regression module to further improve the performance on prognosis prediction.

## Data Availability Statement

The raw data supporting the conclusions of this article will be made available by the authors, without undue reservation.

## Author Contributions

ZY and LL designed the research. ZY, JW, JG, and XX implemented the proposed method and analyzed the data. LL and NC were responsible for the dataset. JW wrote the manuscript. All authors discussed the results and commented on the manuscript. All authors contributed to the article and approved the submitted version.

## Funding

This work was supported by the National Natural Science Foundation of China (grant no. 61906127), the National Major Science and Technology Projects (grant no. 2018AAA0100201), and the Major Scientific and Technological Projects of the New Generation of Artificial Intelligence in Sichuan Province in 2018 (grant no. 2018GZDZX0035).

## Conflict of Interest

The authors declare that the research was conducted in the absence of any commercial or financial relationships that could be construed as a potential conflict of interest.
